# A Data-Driven Approach to Enhance the Prediction of Bacteria–Metabolite Interactions in the Human Gut Microbiome Using Enzyme Encodings and Metabolite Structural Embeddings

**DOI:** 10.3390/nu17030469

**Published:** 2025-01-28

**Authors:** Gopal Srivastava, Michal Brylinski

**Affiliations:** 1 Department of Biological Sciences, Louisiana State University, Baton Rouge, LA 70803, USA; gsriva2@lsu.edu; 2 Center for Computation and Technology, Louisiana State University, Baton Rouge, LA 70803, USA

**Keywords:** bacteria–metabolite interactions, enzyme–metabolite interactions, theoretical (negative) dataset generation, kernel principal component analysis, enzyme encodings, chemical embeddings, chemical metabolism, human gut microbiome

## Abstract

**Background:** The human gut microbiome is critical for host health by facilitating essential metabolic processes. Our study presents a data-driven analysis across 312 bacterial species and 154 unique metabolites to enhance the understanding of underlying metabolic processes in gut bacteria. The focus of the study was to create a strategy to generate a theoretical (negative) set for binary classification models to predict the consumption and production of metabolites in the human gut microbiome. **Results:** Our models achieved median balanced accuracies of 0.74 for consumption predictions and 0.95 for production predictions, highlighting the effectiveness of this approach in generating reliable negative sets. Additionally, we applied a kernel principal component analysis for dimensionality reduction. The consumption model with a polynomial kernel, and the production model with a radial basis function with 32 reduced features, showed median accuracies of 0.58 and 0.67, respectively. This demonstrates that biological information can still be captured, albeit with some loss, even after reducing the number of features. Furthermore, our models were validated on six previously unseen cases, achieving five correct predictions for consumption and four for production, demonstrating alignment with known biological outcomes. **Conclusions:** These findings highlight the potential of integrating data-driven approaches with machine learning techniques to enhance our understanding of gut microbiome metabolism. This work provides a foundation for creating bacteria–metabolite datasets to enhance machine learning-based predictive tools, with potential applications in developing therapeutic methods targeting gut microbes.

## 1. Introduction

The human intestinal tract is home to a diverse gut microbiota, which plays a critical role in maintaining the host’s health and well-being [1,2]. These microorganisms, which include bacteria, archaea, viruses, and fungi, form complex communities that interact with each other and their chemical environment [3]. These interactions are central to the evolution and stability of the gut microbiota, which has co-evolved with the human host [4,5,6]. The gut microbiota thrives by utilizing nutrients derived from the host’s diet and metabolites produced by other members of the gut microflora. Through the degradation of dietary components, gut microbes extract essential nutrients, making them available for themselves and other members of the microbial community [7,8]. This dynamic ecosystem often exists in a state of symbiosis or mutualism, wherein the microbes and the host mutually benefit from each other’s presence, particularly in healthy individuals [9]. However, various external factors can disrupt this delicate balance, leading to a condition known as dysbiosis [10,11]. For instance, certain endocrine-disrupting chemicals, such as the pesticide chlorpyrifos, or artificial sweeteners like aspartame, have been linked to adverse health outcomes, including obesity, type 2 diabetes, and metabolic syndromes [12,13,14]. These compounds have also been shown to induce dysbiosis in the gut, potentially leading to deleterious metabolic effects on the host [11,15,16,17]. Given the crucial role of the gut microbiota in host health, understanding the metabolic processes within the microbial ecosystem is of utmost importance. A deeper comprehension of these interactions could provide critical insights into preventing and managing metabolic disorders and other health conditions associated with gut microbiota dysbiosis [18].

Understanding these processes requires advanced tools capable of modeling the complexity of biological systems, particularly the intricate interactions within the gut microbiota. In this context, graph neural networks (GNNs) have emerged as powerful tools for predicting metabolite consumption and production in bacteria, offering new avenues for exploring metabolic pathways and their implications for health and disease. One example is the prediction of metabolic pathways using a hybrid framework that incorporates graph attention networks (GANs). This approach analyzes compound characteristics, such as molecular structure and composition, to predict the metabolic pathways in which a drug may participate. This facilitates a deeper understanding of drug absorption, distribution, metabolism, and excretion, providing valuable insights into pharmacokinetics and drug interactions [19].

Although the application of transformer-based models to predict metabolite consumption and production in bacteria is still in its early stages, these architectures have been successfully utilized in biomedicine for tasks such as drug sensitivity prediction, metabolite retention, annotation, and modeling of metabolic reactions in humans. For instance, DrugFormer employs gene-knowledge graphs, GANs, and transformer-based language models to predict drug-resistant cancer cell lines and protein targets, aiding in overcoming drug resistance [20]. Similarly, RT-Transformer combines GANs with a 1D-transformer module to predict retention times in liquid chromatography. By learning effective molecular representations from molecular graphs and fingerprints, RT-Transformer enhances metabolite identification across various chromatographic methods, showcasing the versatility of these models in biomedical applications [21]. Numerous studies have investigated the interaction between gut microbes and their chemical environment using ensemble learning methods. For example, ensemble feature selection techniques have been employed to identify microbial biomarkers associated with inflammatory bowel disease (IBD) [22]. Methods such as conditional mutual information maximization, fast correlation-based filter, and extreme gradient boosting have been applied to develop classification models that assist in diagnosing IBD. More recently, researchers have predicted host phenotypes based on gut microbial composition [23] and identified disease-associated metabolites [24]. These approaches demonstrate significant potential for predicting the consumption and production of metabolites by human gut bacteria, offering new avenues for understanding microbial contributions to health and disease.

To further advance our understanding of the underlying mechanisms that regulate the consumption and production of various metabolites by gut microbes, we present a comprehensive, data-driven approach aimed at unraveling these intricate interactions. By systematically analyzing differences in chemical consumption and production across a broad spectrum of bacterial taxa, we seek to identify distinct features that can inform future research and practical applications. Furthermore, we propose a novel methodology for constructing a robust theoretical (negative) dataset, specifically designed based on the dissimilarity of compounds from those in existing experimental datasets. The curated theoretical set is intended to enhance the development of predictive tools that are capable of forecasting the consumption and production of novel chemical compounds by the gut microbiota. These insights will help to elucidate the role of the gut microbiota in host metabolism and potentially reveal novel therapeutic targets. Furthermore, we employ kernel principal component analysis (KPCA) [25] to assess whether dimensionally reduced features of experimental and theoretical sets can retain biological information while yielding accurate predictions. The work provides a fundamental framework for the more sophisticated machine learning (ML) approaches, such as graph neural networks, which have demonstrated superior performance in complex network-based problems. Such predictive tools can be invaluable for expanding our understanding of microbial metabolism and its far-reaching impact on human health. They can also offer potential novel strategies aimed at managing dysbiosis and associated metabolic disorders, making this work a crucial step for future research.

## 2. Materials and Methods

### 2.1. Chemical–Microbe Interactions

Microbial species and their associated metabolites were obtained from NJS16, a literature-curated interspecies network of the human gut microbiota comprising 4483 entries, representing 570 unique microorganisms and host cells [9,26]. Since we wanted to elucidate differences and predict the bacterial metabolism of human metabolites, the three host (human) cell entries were removed in the preprocessing steps, resulting in a dataset corresponding to 567 microbial species, each annotated with specific metabolic labels, namely “consumption” (import), “production” (export), “molecular degradation”, and combined “consumption and production”. To focus on the role of enzymes in metabolite consumption and production, molecular degradation was categorized under consumption category. Additionally, instances labeled as consumption and production were split into separate entries for each process. Subsequently, all bacterial species in the dataset were mapped to the STRING database of known and predicted protein–protein interactions (PPIs) [27] to retrieve the taxon ID for each bacterium, resulting in a total of 312 bacterial species. Information on Gram-stain and pathogenicity for each bacterium was gathered from the BacMap [28,29] and BacDive [30,31] databases.

A molecular weight filter of 50 to 500 Da was applied to metabolites, based on the observation that compounds within this range generally exhibit favorable diffusion properties across membranes, enhancing their bioavailability and subsequent metabolism [32,33]. This filtering approach ensured the retention of biologically relevant molecules containing at least one carbon atom, while excluding compounds that were either too small to retain functional significance, or too large to efficiently diffuse across membranes. As a result, non-carbon-containing molecules, those with molecular weights below 50 Da, and those exceeding 500 Da were excluded. The remaining metabolites were mapped to the STITCH database, which contains known and predicted interactions between chemicals and proteins [34,35], to obtain the STITCH score for each metabolite–protein pair. This process generated a final dataset comprising 2065 instances, representing interactions from 312 bacterial species and 154 unique metabolites. Each instance was labeled as either consumption or production. Physico-chemical properties, including molecular weight, octanol–water partition coefficient (logP), and the number of hydrogen bond donors and acceptors, were calculated using RDKit v2022.09.5 [32,36].

### 2.2. Curation of Metabolite Classes

The initial classification of 154 metabolites into compound classes was performed by manually assigning each metabolite to a category based on information retrieved from PubChem [37]. This manual curation resulted in 10 distinct categories: alcohols, amines, aromatics, amino acids, carbohydrates, carboxylic acid derivatives, fatty acids, nucleosides, steroid derivatives, and vitamins. A residual category, labeled “others”, was also created to encompass metabolites that did not fit into any of the previously mentioned major categories.

### 2.3. Functional Annotation of Protein Sequences Using DeepECTransformer

To functionally annotate amino acid sequences from the 312 bacterial species obtained from the STITCH database, a neural network-based transformer, DeepECTransformer [38], was employed. DeepECTransformer utilizes two prediction engines, a neural network and a homologous sequence search, to extract latent features from amino acid sequences and predict corresponding Enzyme Commission (EC) numbers. This model also provides a prediction confidence score ranging from 0 to 1. For the validation of the bitwise accuracy of DeepECTransformer, a total of 376,076 protein sequences with known EC numbers from 192 bacterial species within our dataset were collected and DeepECTransformer was run to make predictions of the EC numbers for these sequences. The accuracy of these predictions was evaluated using a bitwise accuracy calculation scheme, designed to assess the precision of the predicted EC numbers. First, the prediction accuracy was considered only if the first digit of the predicted EC number matched the first digit of the experimental EC number. If this condition was not met, the prediction was deemed incorrect. Next, the accuracy was calculated based on whether the predicted EC number matched the true EC number from the STITCH database at varying levels of specificity—the first digit, the first two digits, the first three digits, or all four digits. For EC numbers that were partially missing, the prediction was considered correct if the available digits matched between the predicted and true EC numbers. This rigorous validation approach ensured a robust assessment of the reliability of DeepECTransformer in predicting EC numbers for amino acid sequences. DeepECTransformer was subsequently used to predict EC numbers for all sequences from the 312 bacterial species in our dataset, strengthening the functional annotations.

### 2.4. Random Forest-Based Prediction of Enzyme Substrates and Products

To evaluate the effectiveness of EC2Vec and Mol2vec [39] embeddings in predicting the role of a metabolite in a metabolic reaction—specifically, whether it acts as a substrate or a product—we utilized the BRENDA database [40]. The BRENDA database is a comprehensive repository of enzyme-related information, including curated data on substrates, products, enzyme classes, and their associated reactions. By leveraging this resource, we aimed to rigorously test the predictive power of the embeddings, which encode enzymatic and molecular features into numerical representations suitable for ML models. A set of enzymes that either utilize the identified metabolites as substrates or are involved in their production was compiled from the BRENDA database. These enzymes were specifically associated with the 154 metabolites identified in the metabolite–microbe interaction dataset. To ensure that the enzymes collected from the BRENDA database were of bacterial origin, we matched the first three digits of the EC numbers of enzymes from the 312 bacterial species in our dataset with those of the enzymes from the enzymatic reactions listed in BRENDA.

This comparison involved 1922 and 1998 unique EC numbers associated with substrate and product data, respectively, in the Brenda dataset, against 3009 unique EC numbers in our dataset. The mapping revealed significant overlap, with 1907/1922 (99.21%) substrate EC numbers and 1979/1998 (99.04%) product EC numbers present in the metabolite–microbe interaction in our dataset. Given this substantial overlap, we proceeded with the data directly obtained from the Brenda dataset. The mapping process resulted in a positive dataset for the substrate, containing 5469 instances associated with 1922 unique EC numbers, and for the product, comprising 5644 instances corresponding to 1998 unique EC numbers. The positive instances were defined as pairs of metabolites and enzymes where the metabolite is known to be either a substrate or a product of the enzyme. Conversely, negative instances were defined as metabolite–enzyme pairs where the metabolite is neither a substrate nor a product of the enzyme. This was carried out by pairing BRENDA enzymes that were not in the positive sets with the 154 metabolites, and then extracting an equal number of instances as in the positive set. This approach yielded final binary classification datasets containing 10,918 and 11,262 instances for substrates and products, respectively.

Two random forest (RF) binary classifiers, each with 200 trees, were trained using the generated data to predict substrates and products. The classifiers utilized 1024-dimensional EC2Vec embeddings of enzyme numbers and 300-dimensional structural embeddings of metabolites generated using Mol2vec. The RF models were implemented in Python 3.11, leveraging libraries such as scikit-learn 1.0.2, pandas 1.5.0, joblib 0.17.0, and matplotlib 3.3.2. All computations were performed on a High-Performance Computing (HPC) cluster at LSU, featuring 32-core Intel Xeon Platinum 8358 processors and running the Red Hat Enterprise Linux 8 operating system.

### 2.5. Benchmarking Random Forest Models Against kNN

To validate and compare the predictive capabilities of the previously mentioned RF-based substrate and product models, we constructed a dataset containing enzymatic reactions from the EnzyMine database [41]. This analysis aimed to evaluate whether ML models, such as RF-based models, outperform the k-nearest neighbor (kNN) approach in predicting enzymatic substrates or products. To achieve this, we performed a Tanimoto similarity [42] search to compare the 154 metabolites in our dataset with known substrates and products. Simultaneously, a cosine similarity [43] search was conducted to match EC numbers from the BRENDA substrate/product datasets with those in the EnzyMine database. This dual similarity analysis provided a comprehensive framework for benchmarking the performance of RF-based models against the kNN approach, specifically in predicting the enzymatic roles of metabolites. The Tanimoto similarity search identified 46 unique substrates and 45 unique products from the EnzyMine database that matched the 154 metabolites in our dataset. Following this, the kNN method was applied to classify the instances in the substrate and product datasets generated in the previous section. For kNN classification, we used three and five nearest neighbors, determined by ranking instances based on the highest Euclidean distance-based similarity, which combined both Tanimoto and cosine similarity measures. The majority label was then assigned to each instance based on the true labels of the nearest neighbors. This approach enabled a detailed comparison of ML-based predictions with traditional similarity-based methods.

### 2.6. Analysis of Microbe–Metabolite Interactions

To evaluate potential differences in the number of proteins and enzymes interacting with metabolites—either by binding, using them as substrates, or producing them—between experimental data and a random background set, predictions from the RF models were analyzed. Instances from two datasets, strictly consumption and strictly production, were collected for this assessment. A strictly consumption set consisted of 512 instances representing a microbe–metabolite pair with corresponding consumption labels. The consumption dataset included 180 unique bacteria known to consume 79 unique metabolites, and these bacteria were absent in the strictly production set. Additionally, a strictly production set was created, consisting of 61 instances with 13 different metabolites and 35 unique bacteria that were not present in the previous strictly consumption set. As the counter parts of the consumption and production sets, two random background sets were created, one for the strictly consumption dataset and another for the production dataset. To generate instances in random background sets, for each metabolite–bacteria pair in the consumption or production sets, a bacterium from the list of available bacteria not having a microbe in the experimental instance was collected. This implies that for each experimental instance in the strictly consumption set, a microbe was chosen from 179 bacteria, and for the production set, a bacterium was chosen from the list of 34 unique bacteria that were not present in the experimental instance. Then, these new microbes were paired with the metabolite in the experimental instance to create new instances for random background datasets. This dataset enabled the test to see if there were any significant differences between the number of proteins, enzymes binding to the metabolite, and enzymes using the metabolite as a substrate or producing it in experimental set, compared to the random pairings of microbes with metabolites. For this, the Mann–Whitney U Test [44] was performed between the strictly consumption or production sets and the corresponding random sets for the number of proteins, enzymes binding to metabolites, and enzymes utilizing metabolites as either substrates or products.

### 2.7. Curation of Negative Set

Given that the NJS16 dataset provides experimental evidence of microbe–metabolite interactions, each instance within this dataset can be regarded as a positive instance for constructing a classification model. For each positive instance defined as a metabolite–bacterium pair and with a consumption or production label, we excluded metabolites that belonged to the same chemical category (from a predefined set of ten categories listed in Section 2.2) as the metabolite in the positive instance. This step was crucial to ensure that no metabolites resembling the positive instance were included in the negative set for that bacterium. A superset of negative instances was then generated by combining the remaining structurally distinct metabolites to bacteria in the positive sets: consumption (1325 instances, Supplementary Figure S1A) and production (702 instances, Supplementary Figure S1B), where each negative instance consisted of a distinct metabolite–bacterium pair that did not match any of the positive interactions. From this superset, we down-sampled negative sets (1214 negative instances for consumption and 550 negative instances for production), for which the distribution of the STITCH score between the enzymes and metabolites was the same as for the positive sets. A chi-square test [45] was used to find similar distributions for both the consumption and production sets, ensuring a robust negative dataset for subsequent classification models.

### 2.8. Minimum Number of Enzymes for Classification Models

To determine the optimal number of enzymes to be encoded as 1024-dimensional vectors for training a binary classification model, we constructed binary classification datasets for consumption and production, consisting of 5, 10, 15, 20, 40, 60, 80, 100, and 120 enzymes that bind to given metabolites. The enzymes were selected based on the highest STITCH score, sorted in descending order for that metabolite. For instance, in constructing a dataset with five enzymes binding to a metabolite, if a metabolite bound to more than five enzymes, then the top five enzymes with the highest STITCH scores were selected. In cases where fewer than five enzymes were associated with a metabolite, all available enzymes were selected, and the remaining positions were padded with non-enzymes (EC 0.0.0.0). This approach ensured consistent data dimensions for binary classification datasets. For both the consumption and production sets, RF models consisting of 200 trees were created. A five-fold cross-validation was performed, with stratified sampling for each fold.

### 2.9. Dimensionality Reduction Using Kernel PCA

After determining the minimum number of enzymes required to accurately predict the consumption and production of metabolites in the human gut microbiota, a kernel principal component analysis (KPCA) [25] was conducted on both the consumption and production datasets. The analysis aimed to determine if dimensionality reduction could retain the relevant biological information necessary to make accurate predictions. For this purpose, dimensionality reduction using five different kernels in KPCA, namely cosine, polynomial, radial basis function (RBF), sigmoid, and linear, were explored. The classification dataset for consumption contained 15 enzymes and metabolite embeddings, with 15,660 features derived from the combination of 1024-dimensional EC2Vec embeddings for each enzyme and 300-dimensional Mol2vec embeddings for metabolites. Since the number of principal components was limited by the minimum number of either samples or features, for the consumption dataset containing 2539 instances, this resulted in reduced datasets, with features ranging from 8 to 2048. On the other hand, the production dataset consisted of 10,540 features with 1252 instances, which resulted in reduced datasets with feature counts ranging from 8 to 1024. For each of these sets, RF models consisting of 200 trees were created. The five-fold cross-validation was performed, with stratified sampling for each fold.

### 2.10. Preparation of Unseen Data

After training the consumption and production models and their corresponding dimensionally reduced models, we wanted to test their efficacy on unseen data. For this, we curated two sets of unseen data. For the first dataset, we gathered six instances of metabolite–bacterium pairings for which experimental data were not present in our dataset, but for which there was external literature supporting their consumption or production by the microbes in the respective instances. As for the second unseen dataset, we created negative sets for consumption and production instances. The consumption unseen negative set was generated by pairing 13 metabolites from the strictly production set with 180 bacterial species from the strictly consumption set. Instances with microbes having at least one enzyme binding to metabolites were kept as input for the RF model. Using this procedure, 2275 instances were generated for the consumption unseen negative set. Similarly, the production unseen negative dataset was curated by combining 35 metabolites from the strictly consumption set with 35 microbes from the strictly production set, generating 2539 instances. The new instances were considered valid only if there was at least one enzyme in the microbe metabolizing the metabolite.

Metabolite SMILES embeddings and EC number embeddings for enzymes interacting with these compounds were used as input for the consumption and production models. From these models, prediction probabilities and predicted labels were collected. For the first unseen dataset, the prediction probabilities from both the consumption and production models were combined to calculate a consensus prediction. The consensus for each prediction was determined by averaging the probabilities for positive and negative classifications from both models. Specifically, for the consumption model, the consensus was calculated by combining the probabilities of the compound being classified as positive (consumed) and as negative (not produced). Similarly, for the production model, the consensus was based on the probabilities of the compound being classified as positive (produced) and as negative (not consumed). This approach ensured that both the consumption and production models contributed equally to the final predictions.

## 3. Results

### 3.1. Data Collection, Curation and Analysis

**Figure 1:**
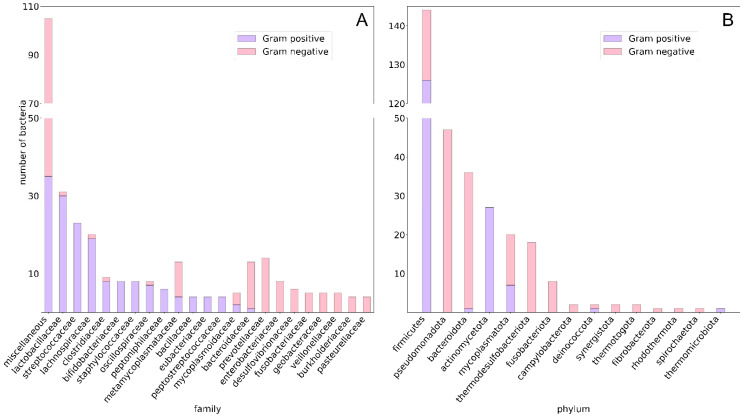
Stacked histograms showing distribution of bacteria across families and phyla for 312 species. (**A**) Number of bacteria per family. Families with fewer than or equal to three species are grouped under “Miscellaneous”. (**B**) Number of bacteria per phylum. Blue bars represent Gram-positive species, while red bars represent Gram-negative species.

To explore the taxonomic diversity of our dataset, we collected the total number of bacteria per phylum and per family. This dataset includes 312 gut bacterial species and 154 unique metabolites, identified by their corresponding compound ID (CID) from the STITCH database [34,35]. These 312 bacterial species are classified across 15 different phyla and 99 distinct families, with an average of 3.15 species per family. Among these, 150 species are Gram-negative and 162 are Gram-positive. Additionally, within the dataset, 105 bacteria are identified as pathogenic to humans and animals, while 111 are labeled as non-pathogenic. Virulence data for the remaining 96 species could not be determined. To streamline the data for analysis and improve interpretability, bacterial families with fewer than three species were grouped into a “miscellaneous” category, resulting in 23 distinct bacterial families being retained for further analysis. Figure 1 provides insight into the distribution of Gram-positive and Gram-negative bacteria across these families, in terms of the number of bacteria per family and per phylum. Figure 1A shows that the “miscellaneous” category consists of the highest number of bacteria in the dataset, with the *Lactobacillaceae* family being the largest specific family, comprising 31 bacterial species. Among Gram-negative bacteria, the *Prevotellaceae* family is the most prominent, containing 14 species. Figure 1B highlights that the *Firmicutes* phylum includes the most Gram-positive bacteria in the dataset, while the *Pseudomonadota* phylum contains the highest number of Gram-negative bacteria. These visualizations provide a clear overview of the taxonomic composition of the gut microbiota represented in the dataset.

**Figure 2:**
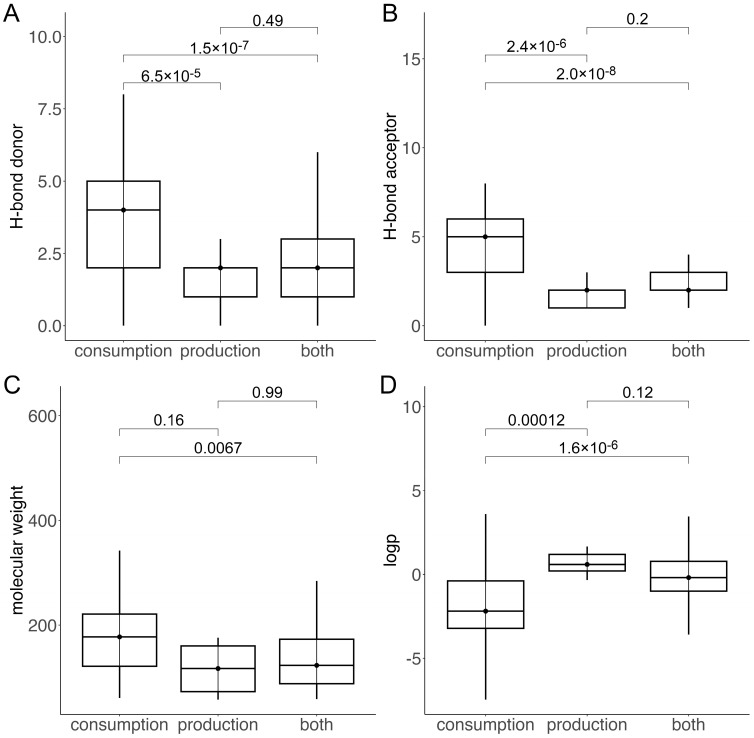
Box plots comparing physico-chemical properties of metabolites across three datasets: strictly consumption, strictly production, and compounds metabolized by bacteria in both consumption and production sets. (**A**) Number of hydrogen bond donors, (**B**) number of hydrogen bond acceptors, (**C**) molecular weight, and (**D**) octanol–water partition coefficient (logP). Horizontal line within each box indicates median value. Significance levels (*p*-values) between distributions are shown at top, with lines indicating datasets being compared.

The analysis of the physico-chemical properties of metabolites across three distinct sets—strictly consumption, strictly production, and a mixed set of metabolites that can be both consumed and produced by bacteria—shown in Figure 2, revealed significant differences between the consumption and production sets. The median number of hydrogen bond donors in the consumption, production, and common sets were four, two, and two, respectively. The median number of hydrogen bond acceptors in the production and common sets was two, while the consumption set had a median of five hydrogen bond acceptors. The median molecular weights of the consumption and production sets were 177.65 Da and 117.15 Da, respectively, with no statistically significant difference between the two. The common set had a median molecular weight of 123.11 Da. The median octanol–water partition coefficients (logP) for the consumption and production sets were −2.19 and 0.59, respectively, while the common set had a median logP of −0.19. This suggests that while metabolites in the consumption and production sets exhibit distinct physico-chemical characteristics, these differences are not as pronounced when considering metabolites that can be both consumed and produced by the microbes in the human gut.

**Figure 3:**
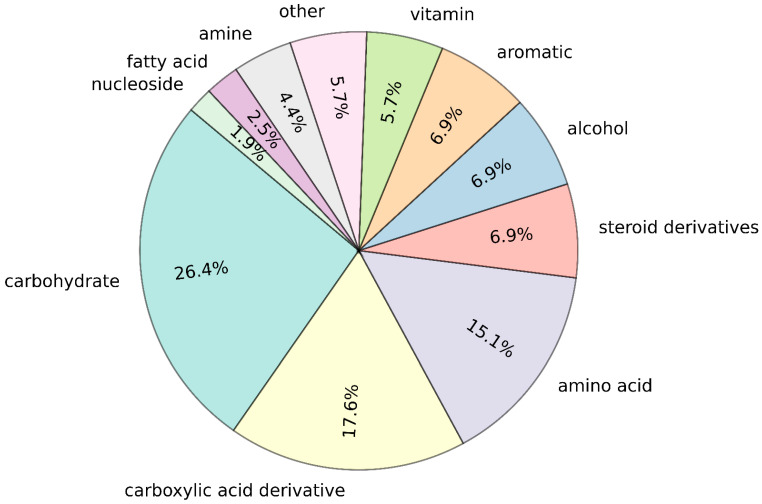
Pie chart showing the percentage distribution of metabolites across ten chemical categories. Each section of the pie chart represents the proportion of metabolites belonging to a specific category. The “other” category includes compounds that could not be classified into any of the nine predefined categories.

The categorization of metabolites shown in Figure 3 reveals that the majority belong to the carbohydrate category, accounting for 26.4% of the total metabolites. Carboxylic acid derivatives and amino acids represent the second and third largest categories, respectively. This distribution is consistent with the biological roles of these compounds, as carbohydrates serve as a primary carbon source for microbes [7], and carboxylic acid derivatives participate in several key metabolic processes. For instance, D-tagaturonate is an intermediate involved in hexuronate degradation in *E. coli*, where it is converted to aldehydo-D-galacturonate [46]. Another example is succinate, a critical metabolite in the tricarboxylic acid cycle, where it acts as a substrate for succinate dehydrogenase, thereby playing a vital role in energy production [47]. This distribution underscores the importance of these metabolites in microbial metabolism and their essential roles in sustaining cellular functions.

**Figure 4:**
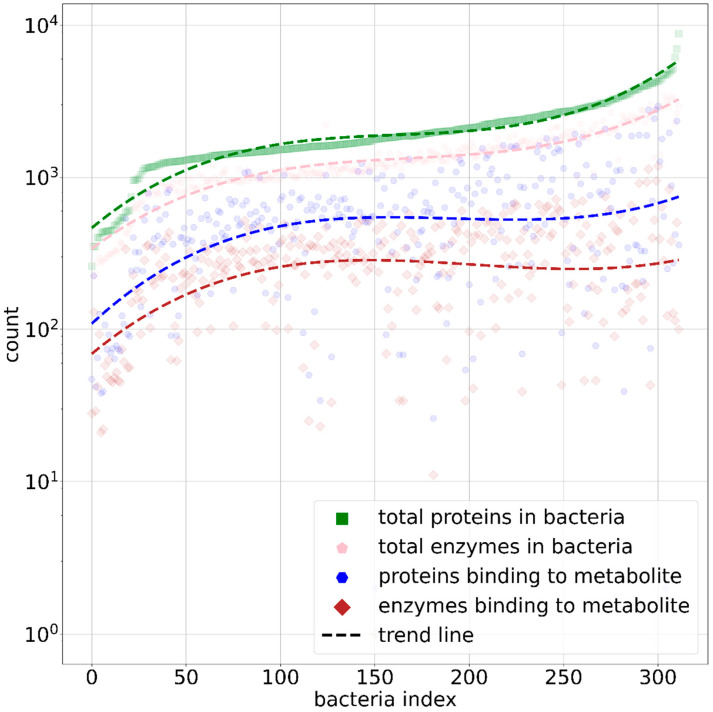
Scatter line plot showing distribution of protein and enzyme counts per bacterium, as well as proteins and enzymes that bind to metabolites. Green squares represent total number of proteins, pink pentagons represent enzymes, blue hexagons represent proteins that bind to metabolites, and brown diamonds represent enzymes binding to metabolites. Dashed lines indicate third-degree polynomial fit for each category. *x*-axis represents bacterial index, sorted by total number of proteins per bacterium in dataset.

In Figure 4, the cumulative distribution of total proteins, enzymes, and metabolite-binding enzymes across the 312 bacterial species in our dataset is illustrated, providing a comprehensive overview of the abundance of these biomolecules within the microbial community under study. In the dataset, 249 bacterial species have a total number of proteins that falls within one standard deviation of the mean protein count. Among these the phylum, *Firmicutes* is the largest, with 132 bacterial species. The mean values for proteins, enzymes, proteins binding to metabolites, and enzymes binding to metabolites are 2050.39 ± 1066.59, 1361.70 ± 624.64, 637.91 ± 514.63, and 298.70 ± 192.43, respectively. Notably, *Mycoplasma haemofelis*, a member of the phylum *Mycoplasmatota*, is the smallest organism in the dataset, with 260 total proteins, 357 enzymes, 47 proteins binding to metabolites, and 28 enzymes binding to metabolites. In contrast, *Burkholderia multivorans*, from the phylum *Pseudomonadota*, represents the largest organism in the dataset, possessing 8800 total proteins, 2952 total enzymes, and 359 proteins and 100 enzymes that bind to metabolites. On the other hand, the *Bacteroides thetaiotaomicron*, which was shown to be most promiscuous bacteria [9], contains 2677 total proteins, 2132 total enzymes, and 1774 and 718 proteins and enzymes binding to metabolites, respectively. In our final curated dataset, there are 30 metabolites consumed and 22 metabolites produced by *Bacteroides thetaiotaomicron.* These values highlight the significant variation in biomolecular content across different bacterial species in the dataset. Given that metabolite conversion is an enzymatic process, the next step was to gather the EC numbers for all enzymes across the microbes in our data.

### 3.2. Accuracy of Functional Annotation with DeepECTransformer

**Figure 5:**
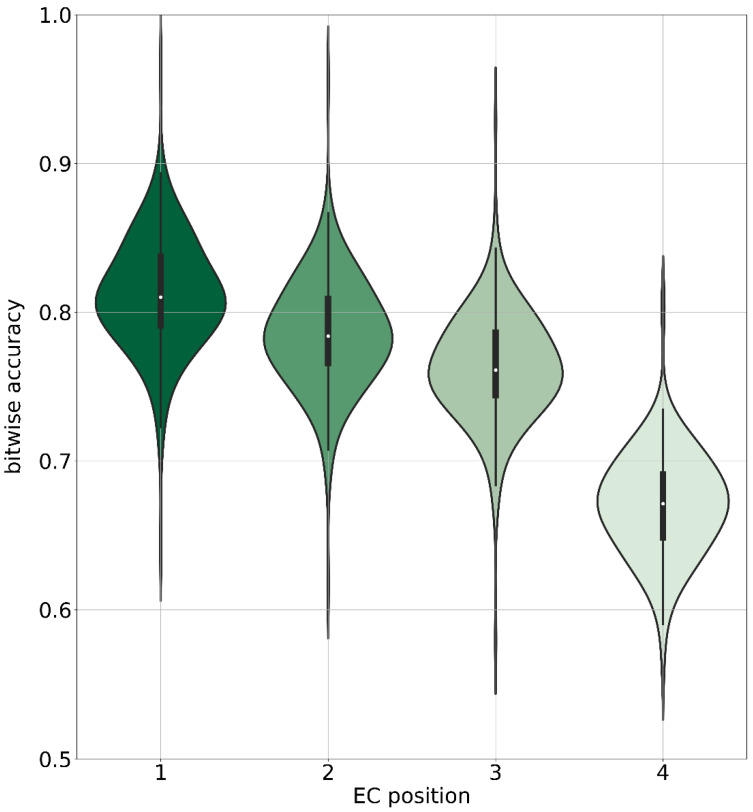
Violin plot illustrating bitwise accuracy of DeepECTransformer predictions for EC numbers on amino acid sequences from 192 bacterial species with experimentally validated EC numbers. White dot in center of each violin represents mean prediction accuracy and different colors represent accuracies at different bit-levels.

The performance of DeepECTransformer was originally reported in terms of F1-scores, which ranged from 0.699 to 0.947 [38]. Here, we conducted an independent validation to assess the accuracy of EC number predictions at different hierarchical levels. Our validation demonstrated bitwise mean accuracies of 0.811 ± 0.037 for the first digit, 0.785 ± 0.037 for the first two digits, 0.763 ± 0.036 for the first three digits, and 0.670 ± 0.034 for all four digits. The distribution of these bitwise accuracies, shown in Figure 5, offers a detailed view of the model performance across different EC number positions. Given the strong predictive accuracy of DeepECTransformer, we utilized this method to predict EC numbers for bacterial sequences obtained from the STRING database [27]. Since the biological conversion of the molecule involves enzymes, we aimed to test whether encoding the EC numbers and SMILES of the metabolites could capture the biological information necessary to indicate their consumption or production. When a metabolite is consumed, it serves as a substrate for an enzyme; conversely, when produced, it acts as a product of the enzymatic reaction.

### 3.3. Feasibility of EC Number Encodings and Chemical Embeddings

To evaluate the feasibility of using EC number encodings from EC2Vec and chemical embeddings from Mol2Vec [39] for predicting whether a metabolite binding to an enzyme acts as a substrate or product, we compiled a dataset of 154 metabolites and their corresponding enzymes. In this dataset, the metabolites function as reactants, either as substrates or products, according to the BRENDA database [40]. The five-fold cross-validated binary classification performance of RF and 3-nearest neighbors (3NN) models for predicting substrates and products is presented in Table 1. 

The RF models demonstrate promising results, achieving balanced accuracies of 0.788 for substrate prediction and 0.791 for product prediction. These outcomes indicate that the embeddings used in these models are effective for predicting metabolite–enzyme interactions. In comparison, the performance of the 3NN classifier is significantly lower than that of RF. Specifically, 3NN-based predictions for the substrate dataset yielded a balanced accuracy of 0.508, while the product dataset prediction shows balanced accuracy of 0.491. These results highlight the superior predictive power of the RF-based approaches over simple deductions relying on cosine similarity with neighboring datapoints. The higher performance of the RF models indicates the effectiveness of enzyme and chemical embeddings in predicting metabolite–enzyme interactions.

**Figure 6:**
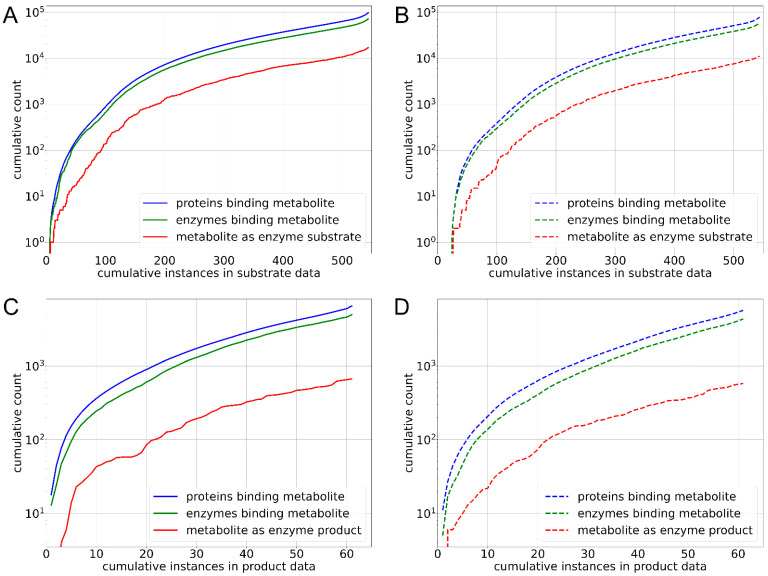
Cumulative histograms comparing number of proteins (blue), enzymes (green), and enzymes utilizing metabolites as substrates or products (red) in experimental dataset (solid lines) versus random background sets (dotted lines). (**A**) Substrates in experimental dataset, (**B**) substrates in random background dataset, (**C**) products in experimental dataset, and (**D**) products in random background dataset. *y*-axis is shown on a logarithmic scale, and *x*-axis represents cumulative instances, indicating total number of instances at specific cumulative counts.

After predicting substrates and products with the models above, we examined the distribution of proteins and enzymes binding to metabolites, focusing on those that use the metabolites as substrates or produce them. Our aim was to determine whether the number of proteins and enzymes observed in experimentally verified instances (metabolite–bacteria label) differed significantly from those in randomly selected bacteria from either the consumption or production sets. Figure 6 shows that the median values in the experimental (consumption) set for the number of proteins binding to metabolites, enzymes binding to metabolites, and enzymes utilizing metabolites as substrates were 136, 98, and 17, respectively (Figure 6A), compared to 100, 76, and 11 in the random set (Figure 6B). Further, we calculated Mann–Whitney U test *p*-values comparing experimental and random sets for three categories: proteins binding to metabolites, enzymes binding to metabolites, and enzymes using metabolites as substrates. The *p*-values for these comparisons were 2 × 10^−4^, 4 × 10^−4^, and 1 × 10^−4^, respectively, indicating statistically significant differences in the number of proteins, enzymes, and enzymes using the metabolite as substrate between the experimental and random sets.

In contrast to the production set, the cumulative numbers of proteins, enzymes binding to metabolites, and enzymes utilizing metabolites as products were comparable between the experimental and random sets. The median values for the experimental (production) set were 95 for proteins binding to metabolites, 80 for enzymes binding to metabolites, and 8 for enzymes producing metabolites (Figure 6C), compared to 76, 57, and 7, respectively, in the random set (Figure 6D). The corresponding *p*-values between the experimental and random sets were 0.24, 0.15, and 0.58, respectively. These findings suggest that while significant differences were observed in the consumption set between the experimental and random sets, no such differences were evident in the case of the production set.

### 3.4. Curation of Negative Set and RF-Based Prediction of Metabolism

Since all the experimental instances in our dataset were positive examples, we needed to create a balanced negative set. To avoid inherent bias in the binary classification dataset caused by metabolite–enzyme affinities, we ensured that the distribution of metabolite–enzyme association scores (STITCH scores) was similar in both the positive and negative sets. We used a chi-square test for this purpose, which yielded chi-square scores of 0.001 and 0.005 for the consumption and production sets, respectively, with a *p*-value of 1 for both, indicating no significant difference. The mean STITCH scores ± standard deviation for the positive and negative instances were, respectively, 389 ± 265 and 371 ± 261 in the consumption set, and 387 ± 274 and 355 ± 252 in the production set (Supplementary Figure S1).

**Figure 7:**
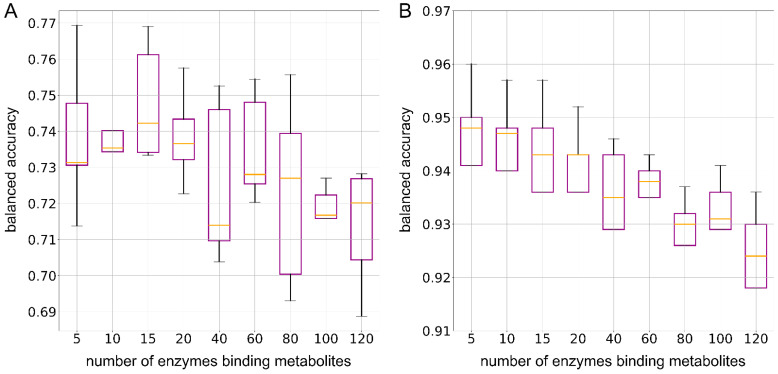
Box plots showing the balanced accuracy of random forest binary classification models as a function of the number of enzymes used to generate enzyme embeddings as features. (**A**) The consumption dataset and (**B**) the production dataset. In each box plot, the orange line indicates the median accuracy.

The next step was to determine the minimum number of enzymes needed to train ML models for the optimal prediction of metabolite consumption and production in human gut microbes. We analyzed enzyme groups ranging from 5 to 120, ranked by their STITCH scores. If an instance did not have the exact number of required enzymes, we supplemented it with encodings from non-enzymes. Figure 7 presents the median balanced accuracy (BAC) achieved by an RF binary classifier trained with varying enzyme group sizes as features. Each group represents a different number of top-ranked enzymes used to predict metabolite consumption and production in human gut microbes. The BAC values illustrate how the choice of enzyme group size impacts classification performance, with specific group sizes leading to higher accuracy in capturing the interactions between enzymes and metabolites. This analysis helps in identifying the optimal number of enzymes needed for accurate predictions. For the consumption set, the classifier achieved its highest median BAC of 0.742 when using the top 15 enzymes as features (Figure 7A). In contrast, for the production set, the optimal model was selected with the top 10 enzymes as features, yielding a median BAC of 0.947 (Figure 7B). This model was chosen because it demonstrated similar mean accuracy to the model using the top five enzymes, but provided a larger feature set, enabling a more detailed examination of how dimensionality reduction affects classification performance in gut microbe metabolism. This additional feature information supports a more comprehensive analysis of enzyme contributions to metabolite production.

### 3.5. Kernel Principal Component Analysis

**Figure 8:**
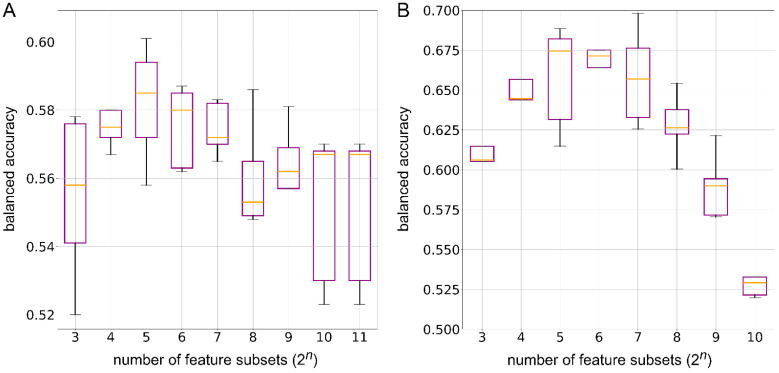
Box plots showing the balanced accuracy of random forest binary classification models trained on polynomial kernel-based dimensionally reduced features, with a varying number of feature subsets (2*^n^*). For the consumption dataset, *n* ranges from 3 to 11, while for the production dataset, *n* ranges from 3 to 10. (**A**) The consumption dataset and (**B**) the production dataset. In each box plot, the orange line represents the median accuracy.

To discern the impact of dimensionality reduction on the biologically relevant encodings, we trained RF models using features with reduced dimensions, ranging from 8 to 2048 for the consumption set. In the consumption prediction, the polynomial kernel showed a median accuracy of 0.582 with 32 features (Figure 8A). The cosine, RBF, linear, and sigmoid kernels showed median accuracies of 0.575 with 64 features, 0.575 with 256 features, 0.579 with 64 features, and 0.581 with 128 reduced features, respectively (Supplementary Figure S2). For the production set, the dimensionally reduced features ranged from 8 to 1024, and the polynomial kernel showed the best median accuracy of 0.674 with 32 features (Figure 8B). The cosine, RBF, linear, and sigmoid kernels demonstrated mean balanced accuracies of 0.668 with 128 features, 0.672 with 32 features, 0.670 with 32 features, and 0.645 with 128 features, respectively (Supplementary Figure S3). These results indicate that ML methods, such as RF, can learn biologically relevant information even with a reduced number of features. Thus, this points towards their relevance in reducing computational cost, with minimal compromise on model performance, in sophisticated ML models such as the GNN.

### 3.6. Validation Against Unseen Data

Following the training of the consumption and production models, we aimed to evaluate their efficacy on previously unseen data. Six test instances were selected: miglitol, betaine, 4-aminobutyrate, maltitol, D-psicose, and taurochenodeoxycholate. For these six unseen cases, the consumption model was able to make five correct predictions, and the production model made four correct predictions. A consensus was calculated based on the prediction probabilities of these two models, leading to five correct predictions with a consensus accuracy of 0.83, which is comparable to previous consumption and production models (Table 2). 

For betaine, 4-aminobutyrate, and D-psicose, both consumption and production models reached a consensus on the production of the consumption of these compounds. In the case of maltitol, the models also reached a consensus, despite the consumption model being unable to provide a specific prediction. Based on this consensus, the consumption of maltitol by *Bacteroides ovatus* was correctly identified. Similarly, for taurochenodeoxycholate, although the production model yielded an incorrect prediction, the consensus between the two models successfully identified the consumption of taurochenodeoxycholate by *Lactobacillus acidophilus*. For miglitol, an α-glucosidase inhibitor used in the treatment of type 2 diabetes [48], the consumption model correctly predicted its production by *Gluconobacter oxydans*, but the production model failed to make a correct prediction.

To further elucidate our predictions and investigate their molecular basis, we analyzed enzymes interacting with compounds in our unseen dataset. Miglitol interacted with two proteins, α-glucosidase (EC 3.2.1.20) and chromosome partition protein (EC 2.3.2.27 and EC 3.6.1). These enzymes did not form a protein–protein interaction, which may account for the incorrect prediction of miglitol production in *G. oxydans* in the consensus results. A consensus on the production of betaine by *Bifidobacterium bifidum* was achieved by both the consumption and production model. Betaine is a naturally occurring choline derivative that is commonly ingested through the diet [49]. The accuracy of the models can be attributed to the EC2Vec embeddings of the enzymes involved in the biochemical pathway of choline to betaine oxidation. For the construction of EC2Vec features, we extracted enzymes binding to betaine from the STITCH database. Betaine interacted with a total of six enzymes in *B. bifidum*, five of which were part of the PPI network. These enzymes were associated with the oxidation of choline by alcohol dehydrogenase (EC 1.1.1.1) and aldehyde dehydrogenase (EC 1.2.1.68), leading to the production of betaine and other compounds, including methionine, homocysteine, and glycine [50].

4-Aminobutyrate (GABA) is an inhibitory neurotransmitter associated with various neurological disorders, including ADHD, Alzheimer’s disease, and autism spectrum disorder [51,52,53]. In the case of the production of GABA, we found 113 proteins in *Bacteroides fragilis* that interact with GABA. Among these 113 proteins, we ranked 15 enzymes with the highest metabolite–enzymes association scores according to STITCH. All of these enzymes, except Xaa-Pro dipeptidase (amidohydrolase) and ThiJ/PfpI family protein, were part of the same PPI network. The enzyme *Glutamate decarboxylase* was part of the network and it was shown to be involved in the production of GABA in *B. fragilis* [54]. On the other hand, maltitol, which is a disaccharide polyol containing D-glucitol with α-D-glucosyl residue [55], interacted with 20 proteins in *B. ovatus*, forming two sub-networks of PPI. *B. ovatus* can utilize maltitol, as well as other sugars like D-arabitol, D-mannitol, and lactitol, to support its growth [56]. In addition to these, it can metabolize peptides, monosaccharides, disaccharides, and polysaccharides [57]. To process these complex molecules, the bacterium requires the activity of peptidases and hydrolases to break them down effectively. Of the twenty proteins in the network binding to maltitol, six were peptidase, forming one of the subnetworks involved in the metabolism of maltitol. The hydrolase was the part of a PPI subnetwork which was linked to enzymes responsible for growth of the organism, including helicase, DNA primase, and DNA polymerase III [34]. Thus, the correct prediction of maltitol by consensus can be attributed to the EC2Vec embeddings generated using these important proteins and enzymes responsible for the growth of the *B. ovatus*.

Both the consumption and production models were able predict the consumption of D-piscose by *Clostridium carboxidivorans*. D-psicose is an epimer of fructose at position C3 which is generally found in commercial carbohydrates and agricultural products [37,58]. In the unseen data, D-psicose, also known as D-allulose, binds to seven different enzymes in *C. carboxidivorans*. This includes sugar-phosphate isomerase (EC 5.3.1.6), which is involved in fructose and mannose metabolism, where allulose is converted to allulose-6-phosphate, later transforming into D-fructose-6-phosphate and participating in the Calvin cycle for carbon fixation [59]. Considering the metabolic pathways linking D-allulose to D-fructose-6-phosphate, there is a possibility that *C. carboxidivorans* can consume D-psicose (D-allulose). For taurochenodeoxycholate (TC) consumption by *L. acidophilus*, the compound was found to interact with a total of 27 proteins in this bacterium, as identified from interactions extracted from STITCH [34]. Among these, 13 proteins were involved in PPIs and drug-protein interactions (DPIs) with taurine and bile acids. The PPI network included two choloylglycine hydrolases, also known as bile salt hydrolases (BSH, EC 3.5.1.24), as well as two alpha/beta hydrolases. Studies have shown that BSHs from *L. acidophilus* exhibit specificity for deconjugating taurine-conjugated bile acids [60], supporting the prediction that *L. acidophilus* is capable of consuming TC.

**Figure 9:**
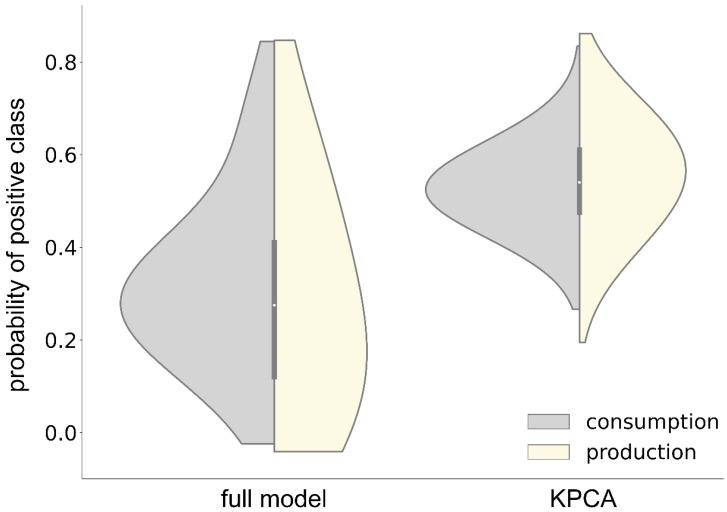
Violin plots showing the positive class probabilities from full and kernel principal component analysis (KPCA) models for consumption and production predictions, validated on unseen negative data. The negative data were generated by combining microbes and metabolites from the strictly consumption and production sets. The light-gray half-violins represent consumption class probabilities from the consumption models (full and KPCA), while the light-yellow half-violins represent production class probabilities from the production models (full and KPCA). The white dot in the middle of the violin represents median probability of prediction.

In the validation conducted on the consumption negative dataset, out of a total of 2275 instances, 1936 instances were predicted as negative, while 339 instances were predicted as positive. Figure 9 shows that the median probability for the positive (consumption) class was 0.29. Similarly, during the validation of the production model on an unseen production negative dataset, 2090 instances were predicted as negative, and 449 instances were predicted as positive, out of 2539 instances, with a median positive class probability of 0.25. Subsequently, we evaluated the KPCA-based models for both consumption and production predictions. The KPCA models yielded median positive class probabilities of 0.52 and 0.55 for consumption and production, respectively. These relatively high probabilities compared to the full models may be attributed to the lower training accuracies observed in the KPCA models within our study. The analysis demonstrates that microbes metabolizing compounds possess proteins and enzymes that form a PPI network, which is directly involved in the metabolic pathways associated with these compounds within the microbial system. This observation supports the rationale for incorporating EC2Vec embeddings in the training of ML models, and provides validation for their predictive performance on previously unseen data.

## 4. Discussion

This study presents a comprehensive data-driven analysis of metabolite–microbe interactions within the human gut microbiome, providing critical insights into the complex metabolic processes that underlie the microbiome in the gut. Given the influence of gut microbes on host health, including their role in various health-related issues depending on host metabolic conditions, this work highlights the importance of curating versatile datasets and biological features for developing predictive ML models. Our results clearly demonstrate that enzyme and metabolite encodings as training features are effective in preserving biological information that is crucial for understanding metabolite–microbe interactions in the human gut. The dataset curated from the NJS16 study, comprising 2065 instances across 312 bacterial species and 154 unique metabolites, provides a robust foundation for exploring the metabolic activities of gut microbes. The taxonomic distribution [61] of these species, primarily within *Firmicutes* and *Pseudomonadota*, is consistent with previous findings that bacteria from these phyla can both be common colonizers of a healthy human gut and that are known to be pathogenic [62,63]. For instance, *Bacillus cereus*, a human pathogen from *Firmicutes*, has been shown to adhere to mucins and alter gut microflora by decreasing populations of *proteobacterium*, like *Escherichia coli* and *Lactobacillus* species [64]. Conversely, *Lactobacillus ruminis*, an indigenous bacterium of the human gut belonging to the *Lactobacillaceae* family, produces lactic acid and helps to maintain a healthy intestinal microflora [65]. Additionally, previously published culture-dependent investigations of the human gut flora have shown that species such as *Bifidobacterium breve*, *B. bifidum*, *B. adolescentis*, *B. pseudocatenulatum*, and *B. animalis* are among the most prevalent in the human gut [66,67,68,69,70]. These members of the *Bifidobacteriaceae* family play a significant role in the gut ecosystem, and have applications in pharmaceuticals and functional food products, due to their ability to exclude intestinal pathogens [71,72,73,74].

Since the human gut microbiome can be affected by the available metabolites in the surroundings, the categorization of metabolites becomes extremely important in understanding metabolite–microbe interactions. Among the metabolites, the prevalence of carbohydrates, carboxylic acid derivatives, and amino acids underlines the central role of these compounds in microbial metabolism, particularly as carbon sources and key intermediates in biochemical pathways. Indeed, the role of carbohydrate-rich diets in contributing to metabolic disorders has been well documented, with these diets also shown to influence the composition and function of the human gut microbiome [75]. Another study demonstrated the impact of carbohydrate ingestion on gut microbiota composition across different taxonomic levels. The findings revealed that soluble fibers increased the abundance of *Bacteroides*, while insoluble fibers were associated with an increase in *Bacteroides* and *Actinobacteria*, and a decrease in *Firmicutes*. Additionally, oligosaccharides were linked to an increase in *Lactobacillus* and a decrease in the *Enterococcus* population within the human gut [76]. These studies further solidify the importance of carbohydrates in shaping the human gut microbiome. Carboxylic acids have been known to be used as food preservatives, and based on the concentration of the carboxylic acids in the gut, they can have inhibitory effects on the bacterial population. A lower concentration of carboxylic acids in the gut environment has been shown to reduce the populations of *E. coli* and *Saccharomyces cerevisiae*, emphasizing the critical role of carboxylic acids and their derivatives in maintaining microbial balance [77]. In some cases, the gut bacteria are responsible for the production of neurotransmitters. For example, a known neurotransmitter, GABA, is produced in the gut by microbes in a higher amount compared to any other human body part [78]. This hints towards differences in the mechanisms of consumption and production of metabolites and their effects on the human gut microbiota.

Since enzymes serve as essential biological catalysts, facilitating the consumption and production of metabolites by accelerating biochemical reactions [79], it is important to study the enzymes found in gut microbes in detail. Given their catalytic role in biological systems, we analyzed the abundance of enzymes in both experimental and random sets. This approach was designed to elucidate bacteria-specific metabolism, recognizing that not all bacterial species possess the same number of enzymes to metabolize a compound, consequently capturing metabolite–enzyme interaction differences at the taxon level. For example, based on the predictions from the BRENDA substrate model, α-ketoglutarate, which was shown to be consumed by *Aeromicrobium marinum* in [80], contained 88 enzymes using it as substrate. On the other hand, *C. carboxidivorans*, from the random dataset, contained only 37 enzymes that use α-ketoglutarate as substrate. In another example from the consumption dataset, *Porphyromonas asaccharolytica* was found to have four enzymes that use 2-oxobutyrate as a substrate [81]. In contrast, *Mycoplasma mycoides*, from the random set, contained only one enzyme utilizing 2-oxobutyrate as a substrate. The analysis showed the variation in the number of enzymes between the experimental and random sets, hinting towards the usefulness of enzyme numbers and metabolite structures in generating features for binary ML classifiers.

Binary ML classifiers require both positive and negative data. Thus, to ensure the robustness of our predictive models, we generated negative sets for both consumption and production, comprising bacterial species and metabolite interactions by removing metabolites that were structurally similar to the molecules in the experimental instances. This process ensured that there was no overlap of compound categories between negative instances and experimental (positive) instances. The negative set simulated a scenario where a bacterium lacks the required enzymes to metabolize certain compounds. The high performance of the RF models for both consumption and production instances underscored the effectiveness of the theoretical negative set. Further, the dimensionality reduction analysis using KPCA revealed that RF models could retain biologically relevant information, even with a reduced number of features. These findings suggest that dimensionality reduction is a valuable tool for optimizing features, reducing computational costs, and preserving biological information, in order to enable the creation of effective machine learning models.

The validation of RF models on the first unseen dataset resulted in five out of six predictions being correctly identified. This high predictive performance can be attributed to the carefully curated input features used in the models. For the second unseen dataset, the results for both consumption and production were as expected. This dataset was generated by cross-combining metabolites and microbes from opposing strictly consumption and production datasets. It is likely that not every microbe in the new negative validation sets possesses the relevant enzymatic pathways required to consume or produce the given metabolites. For example, α-ketobutyrate was predicted as negative for production in *Listeria monocytogenes* by the production model. The prediction can be attributed to the lack of α-ketoglutarate dehydrogenase in the microbe, which leads to incomplete tricarboxylic acid cycle in the microbe [82]. *L. monocytogenes* was predicted to be negative for bicarbonate production. This could be because *L. monocytogenes* consumes bicarbonate to neutralize acidic environments, enhancing its survivability and enabling it to grow across a wide pH range of 4.1 to 9.6 [83].

Literature and database analyses of all the unseen sets revealed that proteins interacting with metabolites in microbes are frequently part of PPI and DPI networks. Among these interacting proteins, enzymes play a pivotal role in metabolic pathways, often catalyzing key biochemical reactions that drive microbial metabolism. For instance, the accurate predictions for metabolites such as taurochenodeoxycholate and 4-aminobutyrate highlight the involvement of bile acid deconjugation and amino acid catabolism pathways, respectively. These pathways are critical for host–microbiome interactions, influencing processes such as bile acid recycling and neurotransmitter regulation. The strong performance of the models also emphasizes the relevance of enzyme embeddings, which integrate enzymatic function and substrate specificity into the predictive framework. By capturing biologically meaningful information, these embeddings likely reflect the functional importance of enzymes in mediating metabolic reactions. Additionally, the predictions align with known metabolic mechanisms, such as sugar fermentation pathways for metabolites like D-psicose and maltitol, further supporting the robustness of the model design. These findings underscore the ability of our machine learning framework to highlight biologically relevant pathways, providing new insights into gut microbial metabolism. Moreover, the effectiveness of the negative set curation strategy strengthens the model’s capacity to predict interactions that align with established biological knowledge, paving the way for novel discoveries in gut microbiome research.

## 5. Conclusions

This study demonstrated the power of combining data-driven approaches with ML techniques to unravel the complexities of the human gut microbiome. By creating a negative dataset and representing metabolite–microbe interactions with enzymatic and chemical embeddings, we were able to establish a framework for future research aimed at accurately predicting the metabolism of compounds by the human gut microbiome. The negative dataset generation strategy can pave the way for the development of novel ML models for therapeutics targeting the gut microbiota, with potential applications in the management of metabolic disorders and other dysbiosis-related conditions.

## Figures and Tables

**Table 1:** Performance of binary classifiers predicting reactants (substrates or products) of enzymatic reactions in gut bacteria, based on BRENDA enzymatic reactions. The classifiers used are Random Forest (RF) and 3-Nearest Neighbors (3NN), with the performance metrics representing the average over 5-fold cross-validation.

Reactant	Classifier	BAC	AUC	PPV	TPR	FPR	F1-Score	MCC
Substrate	RF	0.788	0.870	0.794	0.775	0.200	0.785	0.575
	3NN	0.508	0.508	0.508	0.454	0.438	0.479	0.016
Product	RF	0.791	0.870	0.799	0.775	0.194	0.787	0.582
	3NN	0.491	0.491	0.489	0.479	0.496	0.484	−0.017

BAC—the balanced accuracy, AUC—the area under the ROC curve, PPV—the precision, TPR—the recall, FPR—the false positive rate, MCC—the Matthews correlation coefficient.

**Table 2:** Validation results of random forest models for consumption and production predictions on unseen data. The models were trained using enzyme embeddings derived from 15 EC numbers for the consumption model and 10 EC numbers for the production model. Incorrect predictions are shown in italics, while instances where the consumption model could not make a prediction are labeled “unspecified”. Values under the “Predicted” columns indicate the probabilities for the negative and positive classes in the respective models.

Metabolite	Microbe	Original Label	Predicted Label		
			Consumption Model	Production Model	Consensus
miglitol	*Gluconobacter oxydans*	production	production (0.64, 0.36)	consumption (0.87, 0.13)	consumption (0.62, 0.38)
betaine	*Bifidobacterium bifidum*	production	production (0.62, 0.38)	production (0.48, 0.52)	production (0.57, 0.43)
4-aminobutyrate	*Bacteroides fragilis*	production	production (0.62, 0.38)	production (0.08, 0.92)	production (0.77, 0.23)
maltitol	*Bacteroides ovatus*	consumption	unspecified (0.5, 0.5)	consumption (0.80, 0.20)	consumption (0.35, 0.65)
D-psicose	*Clostridium carboxidivorans*	consumption	consumption (0.27, 0.73)	consumption (0.96, 0.04)	consumption (0.16, 0.84)
taurochenodeoxycholate	*Lactobacillus acidophilus*	consumption	consumption (0.06, 0.94)	production (0.26, 0.74)	consumption (0.40, 0.60)

## Data Availability

Codes and data are available at https://github.com/gnsrivastava/GutMicrobeMetaboliteInteraction accessed on 23 January 2025.

## Supplementary Materials

The following supporting information can be downloaded at https://www.mdpi.com/article/10.3390/nu17030469/s1. Figure S1: Comparison of the distribution of metabolite–enzyme association scores (STITCH scores) between positive and negative sets in the (A) consumption and (B) production datasets. The blue histograms and density lines represent the positive sets, while the orange histograms and density lines represent the negative sets. The vertical dotted lines indicate the mean STITCH scores for the positive sets (blue) and negative sets (orange); Figure S2: Box plots showing the balanced accuracy of random forest binary classification models trained on kernel principal component analysis (KPCA)-reduced features for the consumption dataset, with varying numbers of feature subsets (2n) for different kernel types. (A) Cosine kernel, (B) radial basis function (RBF) kernel, (C) linear kernel, and (D) sigmoid kernel. The value of n varies between 3 and 11 for all kernels except the sigmoid kernel, where n ranges from 3 to 9, due to major eigenvectors becoming negative beyond the 9th component in the consumption dataset. In each box plot, the orange line represents the median balanced accuracy for the random forest model trained with each kernel; Figure S3: Box plots showing the balanced accuracy of random forest binary classification models trained on kernel principal component analysis (KPCA)-reduced features for the production dataset, with varying numbers of feature subsets (2n) for different kernel types. (A) Cosine kernel, (B) radial basis function (RBF) kernel, (C) linear kernel, and (D) sigmoid kernel. The value of n varies between 3 and 10 for all kernels except the sigmoid kernel, where n ranges from 3 to 8 due to major eigenvectors becoming negative beyond the 8th component in the consumption dataset. In each box plot, the orange line represents the median balanced accuracy for the random forest model trained with each kernel.
